# Setting Up a Global System for Sustainable Energy Governance

**DOI:** 10.1007/978-3-030-39066-2_16

**Published:** 2020-03-26

**Authors:** Vladimir Zuev

**Affiliations:** 1 Fondazione Eni Enrico Mattei, Milan, Italy; 2 Fondazione Eni Enrico Mattei, Milan, Italy; National Research University Higher School of Economics (NRUHSE), Moscow, Russia

## Abstract

The global energy landscape is currently shaken by tectonic shifts. We witness dramatic changes in energy geopolitics, the formation of the global system of energy governance, a huge wave of massive technological innovations, global markets are undergoing a radical transformation embracing a fast multiplication of new sources of energy, new products, new producers, and suppliers, coupled with the development of the vast and sophisticated infrastructure and an increasing efficiency in energy use. Each and every component of the system is touched upon by a wind of change that brings about the contours of a new energy global order.

## Energy Geopolitics: From Security Above Anything to Sustainability Among Everything

The global energy landscape is currently shaken by tectonic shifts.^1^ We witness dramatic changes in energy geopolitics, the formation of the global system of energy governance, a huge wave of massive technological innovations, global markets are undergoing a radical transformation embracing a fast multiplication of new sources of energy, new products, new producers, and suppliers, coupled with the development of the vast and sophisticated infrastructure and an increasing efficiency in energy use. Each and every component of the system is touched upon by a wind of change that brings about the contours of a new energy global order.

One core element of the current transformation is evidently the transition from fossil fuels to renewables or rather a rapid rise in renewables usage, especially in those parts of the world which used to be poor in possession of traditional fossil fuels. This transformation will mean a radical shift in the focus of energy geopolitics. Some aspects of this shift have been already mentioned in several studies (Overland 2019). We’d like to outline one more aspect of the new energy geopolitics that seems important for this analysis.

Western countries used to be heavily dependent on oil imports from the Middle East (oil crises of 1973 and 1979), or on gas imports from Russia (Hassanzadeh et al. 2014) (the gas crises between Russia and Ukraine in 2006 and 2009) (Sharples 2016), and on the constantly rising oil prices that reached a peak of $150 a barrel before the global financial and economic crisis. Nowadays, we can register a new phenomenon that we can call a “reversed dependence.” This time, developing countries producing an abundance of fossil fuels are becoming increasingly dependent on consumers from the major developed and emerging economies. The higher the level of renewables will be in the future energy mix the less fossil fuels will be needed globally. If that happens (and there is a high chance it will), this may mean a terrific blow to the developing countries’ energy incomes, affecting many economies and re-shaping geopolitical influences.

The oil-producing countries themselves come to realize all the more, the necessity to diversify their economies away from fossil fuels dependence (OPEC 2019), which could be yet another factor for sustainable economic development. For energy-exporting developing countries that rely on revenues from a limited range of natural resources, the need to reorient their economies is also growing because of an emerging regulatory framework on climate change. Achieving diversification is considered vital for the long-term economic sustainability of their economies. The notion of economic diversification suggests a strategy to transform the economy from using a single resource, or a relatively narrow set of income sources, into multiple sources of income or a considerably broader variety of new and emerging economic sectors. Such a diversification pathway or strategy may be driven by various motivations, with the key objective being to boost economic performance along with sustainable growth.

Energy security, perceived above all as an important element of military and political security, has been one of the top priorities of each and every government of the international community (Blumer et al. 2015). The rapid growth of renewable energy sectors is providing room for a different way of geopolitical thinking, focusing more on economic and sustainability aspects of energy production within, or outside of the national economies, rather than focusing primarily on military and political aspects of energy security at a time of being fully dependent on energy imports (Kelanic 2016; Barnes and Jaffe 2006). The shift can also be traced by using the Energy Trilemma Index of the World Energy Council that is aimed to help countries to formulate better policy through balancing energy security, equity, and environmental sustainability. In other words, the more energy self-sufficient the countries become (most of the renewables can be produced practically everywhere), the better the chances are for sustainable energy development.

It is not only the rise in renewables that is the key to understanding the new system. It is not a mere transition from one energy source to another. In each sector, energy production and consumption become different: more efficient, reliable, safe, affordable and available to everybody, eco and climate-friendly. The bottom-up market and technology-driven transformation of the energy sector, multiplication of energy production sites in each and every corner of the world, have completely revolutionized the way we consume and produce energy. By combining renewable energy, digital technologies, and advanced materials, supported by appropriate infrastructure, the world can modernize the energy system and reduce the flow and the waste of primary resources. Overall, energy usage is becoming, or at least should become sustainable.

## Energy Governance Institutions—A Key to Sustainable Transformation

The market and tech developments represent a basis that is a necessary precondition for the introduction of the new principles into the energy governance. In order to push the economic development in line with the wishful sustainable scenario, one has to have an appropriate system of governance at hand. According to the International Renewable Energy Agency (IRENA) roadmap to 2050, “energy transformation” is possible due to *digitalization, education, and regulation.* We can agree that education is important and not alone in this sector, but in each and every field. Education for new energy generation is imperative. We could add that not only digitalization, but the technological progress in general (batteries, panels, etc.) is also vital for the development of renewables. And the governance challenge that we describe in this chapter, is for sure critical for this transformation.

A sustainable development goal on Energy, formulated as an *access to affordable, reliable, sustainable, and modern energy for all*, has been adopted within the United Nations by governments of 193 countries (UN 2015). The UN set up the foundations, basics for the legal framework of the energy sector development, according to the principles of sustainability and climate protection, as in the UN Convention on climate change, supported by concrete mechanisms provided for in the Kyoto protocol and in the Paris agreement on climate change. The UN has the largest country coverage, as the number of participants is close to totality. However, consensus is hard to reach, decisions are difficult to implement, and big deals are rarely finalized. That is the reason other institutions take their turn to fulfill the mission of global energy governance. We will take a brief look at some of the most important of them for the reasons of the suggested analysis.

*The G20* stands next to the UN in terms of the scope and coverage of the sector, as its members represent about 85% of global GDP. The G20 as an informal institution is quick to react on the burning issues in energy governance, and energy was in focus of most G20 Summits of the recent decade as a high-profile issue. Energy efficiency has been a long-term priority for the G20 as it contributes to the optimum utilization of energy resources. G20 members agree that increased collaboration on energy efficiency can drive economic activity and productivity, strengthen energy security, and improve environmental effects. Energy security, economic efficiency, and environmental safety came to be fully integrated into the G20 sustainable energy development concept.

At the G20 Global Summit on Financing Energy Efficiency, Innovation and Clean Technology in Tokyo, Japan, June 2019, the CEOs of major investment funds and senior financiers joined G20 policy makers, deciding how to close the world’s energy efficiency investment gap. The Summit was organized during the 2019 Japanese Presidency of the G20 in conjunction with the Ministerial Meeting on Energy Transitions for Sustainable Growth. The finance industry debated, with G20 government delegations, on the scope of finance for innovation required to boost the world’s USD 240 billion annual energy efficiency investment market up to one trillion-dollars (G20 2019a). Another priority was how to improve the world´s emerging sustainable energy finance markets. One measure envisaged, is the so called “*green tagging*”—the attachment of energy performance and data to financial performance and data. Increased transparency of the energy performance of banks’ assets through their accelerated tagging could be an additional factor to achieve energy transformation. Green tagging can also serve as an instrument to inform regulators about the impact of energy efficiency on financial activity. At the G20 Global Summit on Financing Energy Efficiency, over 150 high-level delegates concluded a declaration on improving the energy performance of asset investments by financial institutions. They also set up a mode for the implementation of the 2017 G20 Energy Efficiency Investment Toolkit that could support transformation inside member economies. The G20 Energy Efficiency Finance Task Group has developed tools to enable 122 private banks and six public financial institutions, bringing USD 4 trillion for energy efficiency activities. Ministers adopted the “G20 Karuizawa Innovation Action Plan on Energy Transitions and Global Environment for Sustainable Growth,” which will enhance cooperation at national, regional, and international levels (G20 2019b). They also agreed to the initiative “Research and Development 20 for Clean Energy Technologies (RD20),” to promote international collaboration among research institutes in G20 countries.

Though these are mostly recommendations, they are aimed at the point. The G20 Energy Efficiency Leading Program (G20 2016) called for the broadening and deepening of private sector engagement, including through the establishment of a Private Sector Energy Efficiency Investment Platform. Using best practices for energy performance through networks of leading financial institutions is another instrument of the transformation, like the UNEP FI Energy Efficiency Finance Platform. There are many more G20 initiatives, but the ones already mentioned, demonstrate the level of the G20’s engagement to the global sustainable energy governance.

*OPEC* has a special role in global energy governance, as it has an extended mandate and can fix oil production quotas, thus affecting oil prices that can affect all energy prices indirectly. Its goals are well known, and though officially the goals are set to keep prices stable, reasonable, and to reduce price volatility, in reality, they are aimed more to protect the interests of producers in order not to suffer too much from the relatively low price levels, the way these prices happen to be most of the time since the global financial crisis of 2007–09. The deficit of impact on the global production levels, and accordingly on prices, was partially offset by the OPEC+ 11 non-OPEC countries agreement (now +10 as Equatorial Guinea became an OPEC Member in May 2017). Had this been done 5 years earlier, the effect would have been much greater.

Currently, the USA, not being a member of OPEC and not a part of the OPEC+ deal, has become a new important actor on the global energy markets, undergoing a spectacular transformation from a net importer to a net exporter of oil and gas after the shale revolution. The USA alone is capable to compensate for the reduction in the oil production quotas of all OPEC and 11 non-OPEC countries altogether, which downgrades the regulative effects of OPEC’s action on oil production and oil prices. Drone attacks, or the USA—Iran 2020 military tensions, or coronavirus threat from China, do influence more, the volatility of oil prices than OPEC’s actions. The meaning of these consequences is that governing the global security issues has become a factor more important for oil price volatility than energy-related governance per se. Thus, the role of the global institutions like the UN or the G7 could be no less important for global energy governance than the role of specialized energy institutions.

The transformation of the OPEC cooperative framework is very illustrative in a way that it brings together more countries to decide upon the global oil agenda. OPEC+ format becoming permanent is already making OPEC more globalized. Bringing China into the permanent cooperation frame is another change in this direction. Three High-level Meetings of the OPEC-China Energy Dialogue proved to be promising. The third Meeting provided a platform for knowledge exchange, and contributed to the deepening of energy dialogue in general between China and OPEC. Another illustrative change in OPEC’s activities consists of the fact that the sustainability agenda becomes an all the more sensitive and important topic in its work. For OPEC Member Countries and other energy-exporting developing countries that rely on revenues from natural resources, the imperative need to reorient their economies is growing, owing mainly to an emerging stringent regulatory framework on climate change and a sustainable development agenda. Achieving diversification is proclaimed vital for the long-term economic sustainability of the OPEC economies (OPEC 2010, 2019). It remains to be seen how far a strategy to transform the economy from using a single resource, or a relatively narrower set of income sources, into one based on multiple sources of income, or a considerably broader variety of new and emerging economic sectors, will go. For us, it is important to note that such a diversification strategy may be driven by sustainable development concepts.

Energy markets become all the more globalized, like in the case of gas markets that used to be either national or regional connected by the pipe-lines infrastructure. The fast spread of the LNG facilities has led to the fast globalization of the gas markets. As the International Energy Agency notes in its World Energy Outlook, liquified natural gas will surpass pipeline gas as the main way of gas trade over long distances by 2030 (IEA 2019). Within the next 30 years, LNG’s share in total gas demand is projected to rise from 20% in 2018 to 40% in 2040.

Global governance response to these fast changes is slow, and appears to be so far, inadequate. One can’t claim that the *Gas Exporting Countries Forum (GECF)* becomes kind of the new OPEC for the gas markets. Though already in the Doha Declaration, adopted at the first GECF Summit in November 2011, member-states agreed upon the need for fair pricing with respect to a balanced distribution of market risks between gas producers and consumers. However, no robust mechanism to safeguard fair pricing was provided for. The idea of gas prices indexation to oil prices, or support of the long-term gas contracts, put forward at the 2nd Summit in 2013 in Moscow, came into conflict with the fast development of the global gas market with spot sales booming. There is simply no need and it is not realistically feasible to arrange the gas production in a similar way to oil production.

It is interesting to note that the fossil fuel-producing countries put a special emphasis on making evident a link between sustainable development and fossil fuels’ continued production increase to the energy-consuming countries. At the 4th Summit of GECF in Bolivia in November, 2017, the participating countries focused on the promotion of natural gas as an environmentally friendly type of fuel, and on the need for using the potential of natural gas for the implementation of the UN approved Sustainable Development Goals and the Paris Climate Agreement. At the 5th GECF Summit in Malabo (Equatorial Guinea) in November, 2019, the Declaration itself was under-titled “Natural Gas: Energy for Sustainable Development,” to highlight the need to use gas as the core source of energy for Africa. The same focus on sustainability has been noticed through OPEC’s activities (see above). It becomes clear that the future of fossil fuels will depend to a large extent, among other factors, on the regulators’ ability to make fossil fuels usage, eco-friendly, and sustainable. This task is becoming more ambitious under additional pressure from the fast rise in renewables usage, that will serve as an extra point of reference to the efficiency of the traditional energy sources.

Renewables surge can be largely accredited to the regulators’ deliberate policies to push forward sustainable energy networks. An evident example of this kind could be the EU energy policies. *The International Renewable Energy Agency (IRENA)* is another global intergovernmental organization case that supports countries in their transition to a sustainable energy future, and serves as a platform for international cooperation, facilitating technology transfer on renewable energy. IRENA promotes the widespread adoption and sustainable use of all forms of renewable energy, in the pursuit of sustainable development, energy security, and low-carbon economic growth. The adoption of the Agenda for Sustainable Development and Sustainable Development Goals (SDGs), and the Paris Agreement on Climate Change in 2015, provided a powerful global signal for a transition to sustainable energy. Since then IRENA membership doubled to 154 countries and 26 more in accession, with around 1100 governmental representatives, demonstrating the Agency’s rise in global significance for energy governance. The instruments of cooperation are innovative, compared to traditional energy institutions, the way renewables are innovative. The work is based on designing a centre of excellence for energy transformation, making heard the “Global voice of Renewables” and spreading around the “advice and support” initiatives, creating a Network Hub.

The paradox critical assessment of the IRENA’s work could be expressed in a way that it is too much centered upon renewables. The global goal, officially formulated and advanced by IRENA, consists of supporting and fostering the “energy transit” and turning the energy system *from the one based on fossil fuels to another one that enhances efficiency and is based on renewable energy*. The *International Energy Agency’s* (IEA) work, as well as the task of the Agreement on the International Energy Program signed in Paris in November, 1974 are similarly envisaging to reduce dependence on oil and to develop alternative energy sources.

However, in traditional energy sectors from coal to atomic energy, countries, and producers do also improve efficiency and security. For instance, in the OPEC Long-term strategy, adopted 2019, it is stated that “OPEC supports the development and promotion of technologies that advance the environmental performance of oil, and advocates the continuous improvement in standards for exploration and development activities” (OPEC 2010, 2019). Thus, these other traditional energy sectors could also be integrated into the concept of sustainable development, especially having in mind the realistic assessment that their importance will be still great for decades ahead. Though, it is natural that priority in IRENA’s or in the IEA work is given to renewables, denying that much of a role for other sources of energy in the future system could be unnecessary discouraging for many countries and sectors in their efforts toward sustainability.

Many energy organizations that seemed to be far from the sustainable energy concept, are doing a lot, building alliances and shifting their priorities toward sustainable development. For instance, since 1996, IAEA joined the Uranium Group (since then it is called Joint NEA-IAEA Uranium Group). The Nuclear Energy Agency (NEA) itself was set up within the OECD framework, to deal with nuclear power safety. Recently, the IAEA and NEA under the Uranium Group started to coordinate their work to better meet the climate change targets, to advance research and technologies on small modular reactors, upgrading safety standards, and embracing human capital into the concept of nuclear energy development. All of these changes fall well into the concept of sustainable energy governance. Thus, the traditional energy institutions like OPEC, GECF, IAEA are in the process of active adaptation of their respective agendas, to the sustainable development goals that have to be further encouraged by the international community.

## Setting Up a Global System of Sustainable Energy Governance

Global, regional, and national institutions set up a frame and a vector for the energy sectors’ transformation and a top-down policy driven decarbonization, affecting both market conditions and fostering tech innovations, predetermining to a high extent the future energy landscape. We put into focus, global governance institutions and the way they act and should act to make energy transition sustainable. National energy systems are no longer isolated, they are becoming all the more interconnected and interdependent, thus making global governance—an objective imperative. With time, the necessity for coordinated action will be felt as more of a necessity. To make Global Energy Governance (GEG) ready to meet the challenges of the day is becoming an important global policy task. Which mechanisms, at a global level, will help to generate the major changes in a sustainable way? What is missing to make the energy sector sustainable? What model should be chosen to move forward at a global institutions level?

Analyzing the activities of the international energy organizations, we can find a lot of evidence of intensive cooperation links between them either, on ad hoc or on permanent basis, to support the argument of an emerging global energy network. Some cases are listed below.

The United Nations has its special place in the center of the system, setting up the concepts, the goals and institutions, providing a legal basis for energy governance by concluding conventions and agreements, and providing a framework for cooperation between all the major international energy organizations. Advancing the Sustainable Development Goals had a special milestone meaning for the global energy governance, with a particular emphasis to achieve the 7th Goal: “Ensure access to affordable, reliable, sustainable and modern energy for all.” Numerous expert discussions in different formats, like at the Conference of Parties (COP) of the United Nations Framework Convention on Climate Change (UNFCCC), do have a huge capacity-building potential for energy governance.

The G20, G7, OECD, IEA assure the global coordination for sustainable energy. They work together on concepts and ways to achieve the transformation. Since the Pittsburgh G20 Summit in 2009, the IEA has actively contributed to all energy-related activities of the G20, including those on energy security, energy data, market transparency, renewable energy, energy access, energy efficiency, and phasing-out fossil fuel subsidies.

The G20 Summit’s work on financing energy efficiency in June 2019 in Tokyo, was a very illustrative evidence of an intense cooperation between the global general competence organizations and specialized energy institutions. This work was coordinated by the *UN Environment’s Finance Initiative (UNEP FI)* and the *International Partnership for Energy Efficiency Cooperation (IPEEC)*, as co-hosts of the G20 Energy Efficiency Finance Task Group (“EEFTG”). The experts, negotiators, and industry representatives were brought together under the Japanese Presidency of the G20 with the CEOs of major financial institutions, to find a common way forward in energy governance (UNEP Finance Initiative 2019).

The *World Energy Council* is another place where national committees from 100 countries and about 3,000 energy-related organizations, work together promoting the sustainable supply and use of energy. The World Energy Congress, which takes place every three years, is a strategic place for many countries where trends in the rapidly changing energy sector are discussed. In 2019, the congress was held in Abu Dhabi and the Council issued a Report on the “World energy scenarios,” that showed global energy pathways to 2040 in line with the sustainable scenario.

June 7, 2017, IRENA and the State Grid Corporation of China (SGCC), the world’s largest utility company, agreed to enhance cooperation on advancing the energy transition. The International Atomic Energy Agency (IAEA) and IRENA cooperate in the area of energy planning. Collaboration between the IAEA and IRENA was formalized by a Practical Arrangement in 2016. In 2019, the International Energy Agency and IRENA enhanced cooperation between the two organizations by signing a Memorandum of Understanding. The IEA plays an important role in the global energy debate, and co-operates with a broad range of international organizations and forums. It hosted a number of multilateral organizations at its headquarters in Paris, including the Clean Energy Ministerial Secretariat and the Energy Efficiency Hub. The IEA was also the facilitator for the Bio-Future Platform.

Each year, the IEA, the International Energy Forum (IEF), and OPEC, work together in a joint Symposium on Energy Outlooks, which becomes an important part of their working program. The symposium gathers senior analysts and delegates from energy producing and energy-consuming countries, bringing together oil companies, banks, and experts, to discuss the IEA World Energy Outlook and OPEC’s World Oil Outlook. This dialogue is leading to greater convergence in the baseline data. During the 8th IEA-IEF-OPEC symposium, which was held in 2018 in Riyadh, Saudi Arabia, IEA Executive Director Dr. Birol, who began his career at OPEC before joining the IEA, emphasized that a major dialogue between the IEA and OPEC is critical to ensuring global energy security in an environmentally sound and economically sustainable way (IEA-IEF-OPEC 2018).

Sustainable energy agenda became a priority for many organizations that are not directly or solely linked to energy. For instance, the Sustainable Development Working Group of the *Arctic Council* of the eight Arctic states is committed to promoting sustainable development in the Arctic and improving the living conditions of Arctic communities in general. The spreading of green energy in the Arctic region will become one of the main vectors for the Arctic Council work in the coming years. Protecting the Arctic has a special meaning, therefore, energy projects in the Arctic should be agreed upon and supervised more rigorously from a sustainable energy perspective.

The Food and Agriculture Organization (FAO) is one of the IAEA’s partners. Since 1964, the two organizations govern together the Joint FAO/IAEA Division of Nuclear Techniques in Food and Agriculture. The cooperation envisages, among other things, common targets, joint programming, co-funding, and coordinated management.

Global and regional organizations are in close cooperation on sustainable energy issues. In 2011, the IEA and ASEAN formally recognized their ongoing cooperation in energy-related activities by signing a Memorandum of Understanding focused on information-sharing, training, and capacity-building on key energy priorities in the region such as stable and affordable energy supply, power sector development and market integration, the ASEAN Petroleum Security Agreement, and energy efficiency. Another illustrative example of this kind could be the 2018 Agreement between the IEA and the African Union, for a strategic partnership toward a more secure, sustainable and clean energy future for countries across the African continent, through a memorandum of understanding. Eradicating energy poverty is a priority for the IEA, and the agreement will play a vital role in stepping up efforts to achieve secure and sustainable energy for all.

In October 2015 the IEA signed a Statement of Intent with the APEC Energy Working Group at the APEC Energy Ministers Meeting in Cebu, Philippines. This statement builds upon many years of extensive cooperation, and seeks to expand collaboration in areas including energy security, energy data and statistics, renewable energy, fossil fuel subsidy reform, energy market analysis, and capacity building.

Among the new lines of cooperation between global and regional institutions in the sphere of energy, we can mention the recent developments, when for the last year, the GECF and the EAEU have been discussing the models of cooperation. An agreement has been reached on sending, in the year 2020, an invitation to the Eurasian Economic Commission, to take part in the upcoming GECF Summit. These organizations also discussed eventual spheres of cooperation to create a sustainable and transparent energy market with an option to sign a memorandum of cooperation.

We could further continue listing the evidence of intensive cooperation links between the global and regional energy organizations. Having looked through and having analyzed the above-cited cases, it becomes clear that a solid energy cooperation base has been established within a comprehensive format. There are far-reaching goals and concepts proclaimed. Formal and informal energy institutions do actively work and cooperate with each other in an intensive way to form a new energy system, sustainable and viable with a just place for everybody and access to energy for all. Having made the analysis of the activities of the major energy sector institutions, and taking into consideration the increased level of interaction between them, we can arrive to a conclusion that *a system of global energy governance is being actively formed*. We see more coordination at an international level between the policy regulators, more coherence, a higher role of global institutions in policy priorities formulation in general. We can also conclude that this system of *global sustainable energy governance*, has all the chances to be no less solid and important than recognized systems of global trade or financial governance.

## Looking Forward to Sustainable Energy Governance

One method to advance new ideas within global energy governance may consist of bringing for consideration some of the existing mechanisms from the best regional (EU) and global practices (trade and finance). To what extent these can be applied at a global energy governance level remains to be seen. We made a first attempt of this kind in this book.

It appears that the *European Union* provides the best *model* so far, for regional and global energy governance (see chap. 2). The EU has moved in a tremendously dramatic and successful way from the common energy policy, intensified after the Lisbon Treaty, to the Energy Community and Energy Union concepts, all the way up to the recent Green Energy Initiative. The European Union’s clean energy package sets up an ambitious target of reaching 32% for renewable energy’s share in the total final energy consumption, by 2030. It was made with the support of IRENA, which is another demonstration of the intensity and importance of interlinks between the institutions within the system of the global energy governance. The European Green Deal re-announced in December 2019 by the European Commission, seems to be so far, the best comprehensive plan to achieve sustainable development (European Commission 2019a). The EU concept is coming as a global benchmark—a certain guide “how-to advance” a transformation to a prosperous, inclusive, and sustainable economy on the basis of clean energy concept.

It is not only the EU that can provide useful experience of implementing good governance procedures for the global energy governance. About three decades ago, there was another spectacular development at the global trade governance level. Although few drawbacks can currently be registered within this system. Another governance case, which provides a useful reference for us, can be seen by the global financial governance that has been built up within a decade after the global financial crisis. After the global trade and financial governance surge, today comes the turn of the consistent global energy governance system. Both global trade and financial governance so far, are more advanced in the variety of instruments and more efficient in governing the system compared to global energy governance.

Assessing what is missing from the global energy governance initiatives, could be made by a comparative analysis between a respective global energy institution with the energy policies in the EU, and the ones in global trade and global financial governance according to a set of criteria. Before inventing a bike let’s have a look whether we can make good usage of the already existing one. Some of the most important points for good governance are mentioned below.

Availability of the appropriate vision, *ideas, concepts, goals*. These are well advanced in the system of the global energy institutions (WEC, IEA, IEF, G20). The concept of sustainable energy is well advanced in the UN SDGs and in the agenda of major global institutions—G20, OECD, IEA, International energy forum, GECF, IAEA, OPEC, IRENA. In this chapter, we provided concrete examples of the good goals of different institutions which match in a good way with the concept of sustainable development. The focus within these concepts is made on structural shifts that create new patterns of energy investment, production, supply, and consumption, facilitated by technological transformation of economy, data flows, and new infrastructure systems.

Progress in establishing a solid *legal basis* for energy governance was evident (many conventions and agreements already signed). However, the international legal platform for energy governance could be further improved, mainly in the direction of more binding commitments from the stake-holders. Even in the area of atomic energy and IAEA activities, where binding commitments are badly needed, the reality remains that the nuclear security regime “is still a patchwork of voluntary, non-binding, non-transparent national commitments, ad hoc bilateral and multilateral initiatives, and vague legally binding measures that provide no specific standards that states must follow” (FMWG 2013).

Some important agreements, like the European Energy Charter Treaty, were supposed to set up the sound legal basis for cooperation between the energy producing and energy-consuming countries. The Russian Federation planned to be part of this order and signed the Charter. Unfortunately, the Charter was not ratified by Russia. Time went by and the cooperation environment changed dramatically. In May 2019, the European Commission adopted a proposal to modernize the Energy Charter Treaty (European Commission 2019b). The Commission recommended that the Energy Charter Treaty provided stronger provisions on sustainable development and on the energy transition, in line with recent agreements. In the coming years the necessity to either reconsider the existing legal order or to set it up anew, will only grow.

*Monitoring* procedures become a special issue for energy governance. With some exceptions, monitoring mechanisms within the system of global energy governance are either not provided for at all, or not well organized. For instance, OPEC sets quotas for oil production, but there is no OPEC body to monitor compliance with the fixed quotas. It remains under the responsibility of the countries concerned. Under the OPEC+ agreement there is no clear-cut monitoring mechanism at all. The same situation of the weak structures for monitoring is typical for the global emissions quotas. Thinking about the nuclear security, the 20/20 Commission of Eminent Persons recommended that binding agreements should “…give the IAEA a precise mandate to confirm that these (nuclear security) standards are being implemented” (IAEA 2008). One main achievement of the IAEA was the verification of Iran’s nuclear program, re-confirmed in 2019, that allowed the achieving of a diplomatic breakthrough from 2015. A joint comprehensive plan of action followed, the implementation of which remains again under the control of the IAEA. Even with these instruments in place, suspicions and mistrust remain (D. Trump’s Administration claims against Iran in the beginning of 2020 could be provided as proof). This controversial case is highly revealing. Irrespective of the US President’s Administration’s discontent at the results of the work of IAEA inspectors in Iran overall, monitoring could be considered as sufficiently reliable, as all the sites were under control of international inspectors with appropriate reports at hand. Working further on the reliability of monitoring procedures remains a challenge for sustainable energy governance.

*Access to finance* is critical for the success of energy governance. Proclaimed initiatives and goals should be supported by an appropriate financial contribution. The EU experience in encouraging the development of renewables by an extensive financial contribution, both in direct and indirect way, is a good illustration of this success story (see Chap. 10.1007/978-3-030-39066-2_2). So far, the common financial funds for energy systems’ transformation and adaptation are rare to find, though the traditional fossil fuel energy-rich countries in good times of high energy prices and even now, could have created important common funds for common institutions like OPEC, GECF, EAEU, that could have increased their ability to meet the new energy order challenges in a much better way. The financial part of the Kyoto Protocol mechanism was also an important attempt to provide financial backing for the envisaged transformation.

The decisions of the above mentioned G20 Global Summit on Financing Energy Efficiency, Innovation and Clean Technology in Tokyo, June 2019 are a clear recent example of moving in a proper direction. Access to more finance for energy transformation in a sustainable way, is not only a problem for specialized classical and new energy organizations, but also for the general competence global institutions as well. The banking and the business community are all more involved in the energy transformation activity, as the market realities demonstrate to what extent energy efficiency investments or renewables investment, may happen to be profitable with or even without the support of the regulator, which seemed unlikely only a decade ago.

*Transparency* is fundamental for consensus building on energy transformation. Practically all global energy organizations do contribute in one way or another to increased transparency of the system by data collection, spreading of information, preparing reports, undertaking analysis, and working on statistics. Some of them do contribute more by preparing the regular comprehensive reports, like IAE or IRENA. Others make an additional contribution by a joint effort, like in the case of the recent Energy Data Report presented to the G20 to support energy policies that was prepared by IEA in collaboration with IEF and IRENA (IEA 2018). However, some of the data sources are still missing or considered as not sufficiently reliable, like in the case of Iran’s nuclear facilities, or like in the case of the actual oil production facilities, or CO_2_ country related emissions. On the spot missions, the way they are provided for in the IAEA Treaty, could help to fill in the data gap. However, this mechanism is missing both in specialized organizations as in the case of OPEC, or similarly in the general Treaties, like in the Paris Agreement on climate change. More transparency necessitates more trust and vice versa. On the other hand, digital data platforms, modern systems of space tracking, and other technologies on the rise, as well as intensified governmental cooperation, could help filling in the remaining gaps in reliable data collection and verification.

*Extended mandate* of bodies that are set within the structures of international organizations (Secretariats, Commissions, Councils) predetermines to a large extent their governing role to build the future energy order. The downgraded role of common bodies remains the weak point for global energy governance. The big problem is that most of these bodies do not have enough competences to push forward the concepts and ideas of the organizations they represent. Unlike in the EU, most of the global energy governance bodies do have a very limited mandate. These energy organizations do have Councils, Commissions, and Secretariats, but their mandate is far from being so comprehensive and extended, compared to the one of the European Commission—an executive body of the EU, for example. Most structures of the energy organizations do not have the power of legal initiative, they can’t exercise strict control over the policies of the member-states of the organization and they do not have the power of enforcement or arbitration. Among other things, setting up a kind of specialized *Dispute settlement body* for energy governance, the way it was useful in trade within the WTO for many years before the current deadlock of trade wars, seems to be a helpful move.

One can ask if this is rational or feasible at all for the energy organizations to have a structure more or less similar to the EU structure? However, if we consider the efficiency criteria and if we accept the fact that the EU energy policies could serve as a model for other institutions, then the mandate of the other organizations should be further extended.

*Decision*-*making procedures* represent yet another focal element for the success of any governance system. Most of the energy governance organizations do have consensus ruled systems. Reaching consensus for many countries is extremely difficult, especially when you have as members, countries from different continents, with different traditions and policy priorities with developed or developing economies and not least—energy abundant or energy-dependent economies. Thus, their interests differ to a large extent, and finding consensus is difficult. It makes the governance system slow to advance new decisions needed for the world. Consensus is easier to achieve for ecology and climate change, as awareness of urgency and utility of action is higher in these respective areas. Though, we know how difficult it was to reach consensus at the time of Kyoto protocol, and more so during the Paris agreement. It is still more difficult to reach universal agreement on decarbonisation paths. Some actors will not share the mere idea of decarbonization to the extent other countries will consider that necessary.

Simple majority or qualified majority decision-making prevails in the EU, and that is the reason that member countries are able to make so many important decisions for economic cooperation between them. Moving away from consensus and organizing the redistribution of votes between countries within the organization according to, and in proportion with certain criteria, is not only the reality of the European Union. For instance, in the IMF the voting power depends upon the financial contribution to the Fund. It is true that certain decisions in the energy organizations are also not made by the consensus rule. For example, the decision to accept a new member into OPEC could be passed with a three-fourth majority vote. That is a way forward to increasing the efficiency of decision-making in many global energy organizations (and probably not only in the energy sector). Not all (that is not realistic), but at least some decisions on specific issues relevant for the daily operation of an organization should be passed on a majority vote procedure. That is becoming more of a necessity, taking into consideration that more energy-ecology-climate related problems require faster, if not immediate solutions, and waiting a long time to reach a consensus would be unacceptable where a decision is desperately needed.

*Technology transfer* lies at the heart of the global energy sector transformation. Many organizations and conventions aim to support the increase in energy sustainability by means of technological exchange. That is clearly a distinctive feature of the emerging system of global energy governance. The Kyoto Protocol, the GECF, the IEA, the IEF, the IAEA, the IRENA, and even OPEC, all of the existing energy-related conventions and energy organizations do support, in an open and clearly defined way, the transfer of advanced technologies as an important element to arrive to a sustainable economy on the basis of sustainable energy. October, 2017 at the 2nd General Conference of the IEA on technological cooperation, the main focus was to identify opportunities for cooperation between the states in the exchange. The World Energy Council’s annual report on Innovation insights is an important toolkit for countries wanting to navigate through the fast-changing innovation landscape. From a tech point of view, it becomes feasible and clear, how to make the transition. For instance, the IEA 2019 Report with guidance for policymakers on how to accelerate the decarbonization of the power sector, shows a clear way forward to sustainable energy. An additional point for consideration is the technical assistance procedures that will facilitate the transfer.

If there is a political will, the financial ability (see above) and the technical feasibility for the transfer, the one major remaining issue largely concerns the appropriate protection of intellectual property rights. If the matter relates to trade, there are mechanisms within the WTO for the protection of intellectual property rights. However, the multilateral trade governance system is currently in a mess. Does that mean that the global energy governance should depend upon the system of global trade governance? There should be a link, for sure. On the other hand, there should be some institutions within the energy governance system to deal with the specific issues of protection of intellectual property rights. Alternatively, an intensified cooperation with the WIPO with appropriate legal arrangements could be an important element of the secure transfer of advanced energy technology. The ambition could be not dealing only with the one-way transfer of technologies from the West toward the East, but increasingly on deals the other way around. Organizing the transfer of technologies and safeguarding the protection of the related intellectual property rights in a sound way, could be considered as a fundamental element of the emerging system of sustainable energy governance. The usage of the best practices and an exchange of information in this regard is already strong and supported by many energy organizations (IRENA, IEA).

Technical progress will make it possible to advance new secure and sustainable standards that humanity never dreamed of. Hydro-power and nuclear power stations became that much more advanced that a small-scale production becomes cost-efficient. Will we live to see the time when new global standards will set the maximum size for the hydro or the nuclear power plants, in order to minimize the environmental damage in event of the natural disasters, for instance? Bringing different security concepts together in an integrated global sustainable vision for the technically advanced new energy order becomes a realistic scenario under certain assumptions of the globalization of energy innovations.

*Compliance and enforcement* are at the heart of another critical issue for governance. Enforcement procedures, which are strong in the EU, are practically non-existent in the global energy governance institutions, and agreements (OPEC, GECF, G20, Paris climate). The system relies on nationally driven systems. Though the legal enforcement procedures and instruments of common control are weak in most global energy organizations with rare exceptions (IAEA), stakes for non-compliance are too high. Be it the consequences of non-compliance for climate change, or for nuclear safety, or for security of energy supplies, or for stability of energy prices. In the case of non-compliance with a tariff line in trading metals, or in another case of not abiding fully with the WTO rules of origin for a product, a country or a company can miss million or even billion-dollar deals. In the case of not-respecting the emissions’ quotas under the Paris agreement, or the OECD or IAEA nuclear safety standards, humanity risks not only money, but people’s lives. This situation of high stakes and high risks of energy governance non-compliance puts additional pressure on governments and companies, and puts forward the importance of a responsible attitude to energy usages major decisions. Thus, the chances for higher compliance on critical energy issues depend not only upon procedures and mechanisms within the energy organizations, but also to a large extent on social, moral, and ethical aspects of high-level officials in charge of the energy agenda. Conclusions on this point are left for the readers.

Looking at the present system of energy governance one can suggest many more things to come. According to the IRENA report “Global energy transformation—roadmap to 2050”, “*The challenge that policy makers around the world face, is how to accelerate the transition. Transitioning from a global fossil*-*fuel powered energy system, built*-*up over several hundred years, to one that is sustainable, will require a much greater transformation than current policies envisage*” (IRENA 2018). We fully share this opinion and will continue the efforts to suggest ways and means for the transformation, to the benefit of all people concerned.

## Footnotes

1 The study was supported by the World Economy and International Politics Faculty of the National Research University Higher School of Economics, with a contribution from Frolova K., Bukanova D., Ermolaev D.
